# Effectiveness of a Theory-Based Digital Animated Video Intervention to Reduce Intention and Willingness to Sext Among Diploma Students: Cluster Randomized Controlled Trial

**DOI:** 10.2196/48968

**Published:** 2023-10-20

**Authors:** Norain Mansor, Norliza Ahmad, Salmiah Md Said, Kit-Aun Tan, Rosnah Sutan

**Affiliations:** 1 Department of Community Health Faculty of Medicine and Health Sciences Universiti Putra Malaysia Serdang Malaysia; 2 Ministry of Health Melaka Malaysia; 3 Department of Psychiatry Faculty of Medicine and Health Sciences Universiti Putra Malaysia Serdang Malaysia; 4 Department of Public Health Medicine Faculty of Medicine Universiti Kebangsaan Malaysia Cheras Malaysia

**Keywords:** sexting, randomized controlled trial, YouTube, intention, willingness, young adult, Malaysia, diploma students, digital content, digital health intervention, attrition rate, primary outcome, sexual risk, sexual health, WhatsApp

## Abstract

**Background:**

Sexting refers to the exchange of sexually explicit digital content in the form of texts, photos, or videos. In recent years, sexting has become a public health concern. Surveys in Malaysia show a high prevalence of young adults engaged in sexting. Given that sexting is associated with sexual risk behavior, cyberbullying, and mental health issues, this behavior needs intervention to alleviate the resulting public health burden. However, there is a scarcity of theory-based intervention programs on the prevention of intention and willingness to sext among young adults.

**Objective:**

This study aimed to develop and implement a sexting intervention module guided by the prototype willingness model (PWM), delivered using web-based animated video, and evaluate its effectiveness among diploma students from a public higher educational institution. The primary outcomes were intention and willingness to sext, while the secondary outcomes were knowledge, attitude, perceived norms, and prototype perceptions of sexting.

**Methods:**

This 2-armed, parallel, single-blinded cluster randomized controlled trial was conducted in a public higher educational institution in the state of Melaka, Malaysia. Diploma students from 12 programs were randomly allocated into intervention and control groups. Both groups answered a self-administered web-based questionnaire assessing the outcomes at the baseline. The intervention group received a newly developed intervention module based on the PWM in the form of 5 animated videos posted on a private YouTube platform, while the control group was put on the waitlist. The intervention group was encouraged to discuss any issues raised with the researchers via WhatsApp private chat after viewing the videos. All participants were observed immediately and 3 months postintervention. Data analysis was performed with SPSS (version 26; IBM Corp). A generalized linear mixed model was used to determine the effectiveness of the intervention.

**Results:**

There were a total of 300 participants with an attrition rate of 8.3% (n=25). After adjusting for age, sex, relationship status, and the amount of time spent on the web, there were significant differences in the intention to sext (β=–.12; *P*=.002; Cohen *d*=0.23), willingness to sext (β=–.16; *P*<.001; Cohen *d*=0.40), knowledge (β=.12; *P*<.001; Cohen *d*=0.39), attitude (β=–.11; *P*=.001; Cohen *d*=0.31), perceived norms (β=–.06; *P*=.04; Cohen *d*=0.18), and prototype perceptions (β=–.11; *P*<.001; Cohen *d*=0.35) between the intervention and control groups over 3 months.

**Conclusions:**

In this study, the sexting intervention module using the PWM that was delivered via web-based animated videos was effective in reducing intention and willingness to sext as well as in improving knowledge of sexting, attitudes, perceived norms, and prototype perceptions. Therefore, relevant agencies involved in the promotion of sexual and reproductive health among young adults in Malaysia can consider the implementation of this module.

**Trial Registration:**

Thai Clinical Trial Registry TCTR20201010002; https://www.thaiclinicaltrials.org/show/TCTR20201002001

## Introduction

### Background

Sexting refers to the exchange of sexually explicit digital content in the form of texts, photos, or videos [1,2]. Sexting may occur in a variety of contexts, whether during web-based conversations with a romantic partner or while communicating with a stranger via web-based applications [3,4], and it has been proposed as a precursor to premarital sex [5]. In Malaysia, this behavior is deemed inappropriate and unacceptable [6]. In addition, the sharing of obscene content via media is punishable under Malaysian law, whereby the punishment would be more severe if minors were involved [7,8].

In recent years, sexting has emerged as a major public health concern in Malaysia. According to the literature, the prevalence of sexting among Malaysian youths was higher than in Western nations [1,9,10]. Almost three-quarters of young adults in Malaysia admitted that they had sexted before, based on a purposive web-based survey [10]. In addition, a cross-sectional study conducted among 1134 Malaysian youth reported that the prevalence of sharing digital pornographic materials with friends was 17% [11]. Considering the high prevalence of sexting among youths in Malaysia, it is imperative to identify targeted interventions that can address sexting-related issues.

Moreover, sexting has been significantly associated with sexual risk behavior, cyberbullying, and mental health problems [12-16]. Highlights from past studies include a meta-analysis that showed that youths who were involved in sexting were more likely to have engaged in sexual activity (OR 3.66, 95% CI 2.71-4.92) and have multiple sexual partners (OR 5.37, 95% CI 2.72-12.67) [14]. A study conducted among undergraduate students in the United States has reported individuals who were engaged in sexting were 3 times more likely to have unprotected sex for the past 3 months as compared to those who do not engage in sexting (OR 2.97, 95% CI 2.12-4.16) [15]. In addition, a survey among 712 university students in Malaysia found that 66% of them have experienced cyberbullying, with nearly half of those affected experiencing psychological problems [17]. Worse still, a small number (1.5%) have attempted suicide [17]. Due to the profound impact of sexting and its association with other risky behaviors, sexting must be curtailed in Malaysia to minimize the undesirable effects on young people’s health.

### Prior Work

To date, several observational studies have asserted that the behavior of sexting is preceded by an intention and willingness to sext [4,18,19]. Intention refers to a deliberately planned behavior [20], while willingness refers to an individual's spontaneous response to risky circumstances [21]. Both intention and willingness are closely involved in the cognitive process that precedes a certain behavior [22]. By focusing on interventions targeting the intention and willingness to sext, it is possible to prevent sexting among those who have never tried, reduce sexting among those who have sexted, and mitigate potential risky behaviors associated with sexting. In this study, we used the prototype willingness model (PWM) to guide the development of an intervention that included attitude, perceived norms, and prototype perceptions in its constructs [4,19,23-27].

In this digital age, animated videos are widely acknowledged as an effective teaching tool, especially for students in higher educational institutions (HEIs) [28-30]. On a similar note, they have also been shown as excellent educational tools for sexual and reproductive health (SRH) topics for youths [31-33].

### Objective

This study aimed to develop, implement, and evaluate the effects of the newly developed sexting intervention module (SIM) delivered via web-based animated videos on the intention and willingness to sext among diploma students. We hypothesized that the intervention would reduce the intention and willingness to sext among diploma students. Moreover, it would improve the knowledge, attitude, perceived norms, and prototype perceptions of sexting among participants.

## Methods

### Study Design

This study was a 2-armed, parallel, single-blinded, cluster randomized controlled field trial. The available program on the main campus was chosen as a cluster unit with the assumption that students from a specific program only interact with those within the same program as they only engaged in web-based learning at home and other curricular activities were restricted during the COVID-19 pandemic. The intervention arm received SIM, whereas the control arm received a standard information program provided by the HEI and was put on the waitlist.

### Setting and Recruitment

This study was conducted in one of the public HEIs in Melaka state, Malaysia. Melaka state was selected as the study site as it is one of the cities in the Association of Southeast Asian Nations that has committed to achieving 0 new HIV infections by 2030 [34]. More importantly, Melaka recorded a drastic surge in gonorrhea cases from 3 per 100,000 population in 2012 to 11.4 per 100,000 population in 2017, with the majority of the cases occurring among people aged 19 to 30 years [35]. Furthermore, 6% of new HIV cases were also reported during the same period among students from HEIs in Melaka [35]. Thus, this study was deemed to be important in raising awareness about sexting-related issues in Melaka.

The study was conducted on the main campus of the largest HEI in Melaka, which is situated in the Alor Gajah District. The campus offered 13 diploma programs and a bachelor’s program. The inclusion criteria were diploma students, aged 18 to 24 years, unmarried, full-time students, and Malaysian citizens. Students who were on medical leave or deferment and students without access to the internet were excluded.

### Randomization and Allocation Concealment

The 12 programs on the main campus were randomly allocated into intervention and control groups at a ratio of 1:1. All programs were number-coded, and subsequently, a simple randomization process was conducted using Random.Org software (Random.Org LLC) [36]. During participant recruitment, all participants were informed that an intervention would be offered. Therefore, they were not aware of any group allocation throughout the study. Participants were blinded as their awareness of participation in the control group could influence their responses to the questionnaires. This task was performed by a research assistant who was not part of this study. The researcher was also unaware of the group assignment until after the randomization was completed. Participants from each program were selected using proportionate stratified random sampling. Those considered eligible and consented to participate were recruited in the study.

### Sample Size Calculation

The sample size was calculated to detect a mean difference of 0.44 (SD 2.19) in an intention toward sexting between the intervention and control groups based on the closest variable to this study [37]. The significant α level was set at .05 with 80% power, while a fixed number of cluster formula were used for sample size calculation [38]. The cluster number (k) was fixed at 12. The coefficient variance for the cluster size and the intraclass correlation coefficient was set at 0.4 and 0.05, respectively [39]. The required sample size per arm using individual randomization formula calculation to fit in the formula for a fixed number of clusters was also calculated [40]. Finally, the total sample size calculated derived from the formula was 250. However, considering the 20% attrition rate, the overall sample size was 300 with an average cluster size of 25. Subsequently, a total of 150 respondents were required for each intervention and control arm using a 1:1 ratio allocation.

### Intervention Module

The newly developed intervention module, SIM, used 3 constructs of PWM, including attitude, perceived norms, and prototype perceptions. It was developed in Malay, which is the local native language. The module consisted of information regarding the definition of sexting, the negative impact of sexting, the prevalence of sexting, people’s opinions about sexting, and information regarding the negative characters of a sexter (person who sexts). In addition, behavior change techniques were also incorporated in the module, including the use of prompts to identify risky situations and barriers as well as the provision of specific instruction to prevent sexting, to enhance the effectiveness of the intervention [41]. The intervention module was converted into 5 animated videos with a total duration of 24 minutes. The animated videos were pretested among 30 diploma students who were not included in the main study. Its content was validated by public health experts and clinical psychologists in terms of its objective, structure, presentation, and relevance using an assessment tool [42]. Following that, the intervention was delivered to the intervention group using a private YouTube link for 3 subsequent days. Only participants in the intervention arm were granted access to view the animated video. The intervention group was reminded not to share the videos with their peers as these videos were still under trial, and it was important for the study that the participants adhered to these instructions. The intervention was delivered by the primary researcher, who is a physician. Intervention participants were encouraged to discuss with the researcher via WhatsApp private chat any issues raised after viewing the videos. A summary of SIM can be viewed in Multimedia Appendix 1. A detailed description of SIM was described elsewhere.

### Outcomes

#### Overview

A validated self-administered questionnaire was used to measure the sociodemographic characteristics and the outcomes of this study. The questionnaire was divided into 2 sections. The first section captured the respondent’s age, sex, relationship status, and the amount of time spent on the web. The questions in the second section measured the primary and secondary outcomes. The primary outcomes were intention and willingness to sext, while secondary outcomes included knowledge, attitude, perceived norms, and prototype perceptions.

#### Primary Outcome Measures

The intention to sext was measured using a 15-item questionnaire with a 6-point Likert scale adapted from a previous study [26]. The responses ranged from 1 (strongly agree) to 6 (strongly disagree). The reported Cronbach α was .87. The willingness to sext was measured using a 12-item questionnaire on a 6-point Likert scale response [19] that ranged from 1 (certainly not) to 6 (certainly yes). The reported Cronbach α was .93.

#### Secondary Outcome Measures

An 8-item questionnaire was used to measure the knowledge of sexting with multiple-choice responses: “yes,” no,” or “I don’t know” [1,2,15,43]. A score of 1 was given for a correct answer, and a score of 0 was given for an incorrect answer. The Cronbach α reported for this scale was .70. Attitude toward sexting was measured using a 22-item questionnaire with a 6-point Likert scale response adapted from a previous study [31] with a Cronbach α of .83. The perceived norms toward sexting was measured using 16 items from 2 subscales adapted from previous studies [19,44] that reported a Cronbach α of .89. Lastly, the prototype perceptions toward sexters was measured using a 9-item questionnaire [4] with a reported Cronbach α of .81.

### Data Collection

Data were collected at 3 different time points, namely, baseline, immediate postintervention, and 3 months postintervention starting from December 2021 to May 2022. Due to the COVID-19 pandemic, data collection was performed via a web-based Google form. The link was sent to all the participants, and they were given 1 week to submit their responses. Reminders were sent to participants who had yet to submit their responses.

### Data Analysis

Data analysis was performed with SPSS (version 26.0; IBM Corp). The participants were analyzed based on their initial assigned group. Data cleaning and error checking were performed before analysis. Median and IQR were used to summarize the nonnormally distributed data for continuous variables, while frequencies and percentages were used to report the categorical variables. The comparison of baseline characteristics between the 2 arms was conducted using the Mann-Whitney test for continuous variables and chi-square for categorical variables. A generalized linear mixed model (GLMM) was used to assess the group, time, and group and time interaction effect on the intention and willingness to sext, knowledge, attitude, perceived norms, and prototype perceptions between the 2 groups before and after controlling for covariates (age, sex, relationship status, and the amount of time spent on the web).

### Ethics Approval

This study was approved by the Ethics Committee for Research Involving Human Subjects Universiti Putra Malaysia (JKEUPM-2020-321). The HEI administration and head of program approval as well as the respondents’ written consent were obtained before the study.

## Results

### Participants’ Details

**Figure 1:**
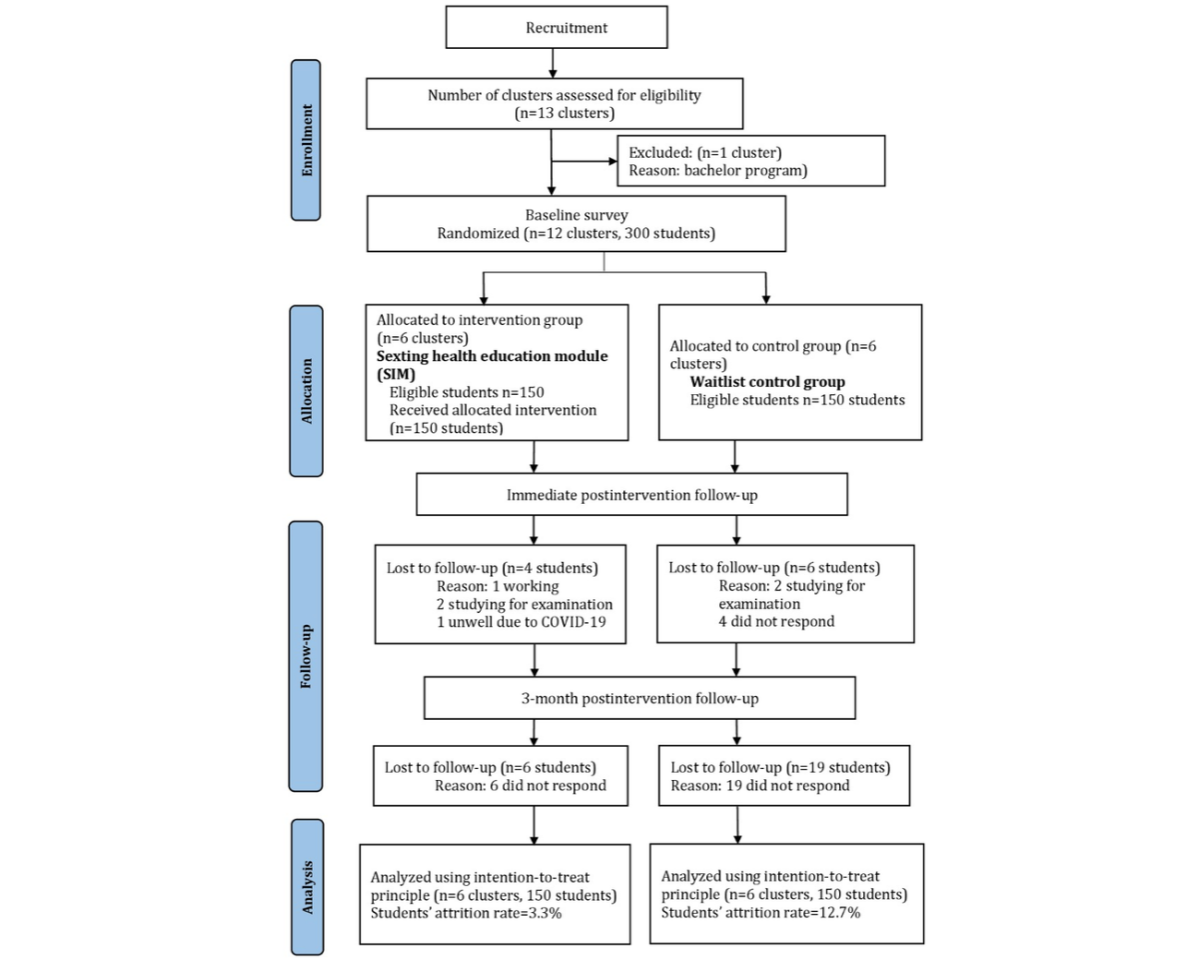
Flowchart diagram of cluster randomized controlled trial on sexting intervention module among diploma students in a public university in Melaka, Malaysia.

A total of 300 eligible participants (intervention group: n=150 and control group: n=150) were recruited for this study in December 2021 and completed follow-up in May 2022. The overall retention rate at 3 months postintervention was 91.7% (275/300). Figure 1 illustrates the Consolidated Standards of Reporting Trials (CONSORT) flowchart of the study (Multimedia Appendix 2).

At baseline, there were statistically significant sex and age differences between the intervention and control groups (Table 1). A total of 8% (25/300) of the data were missing completely at random (*χ*^2^_36_=36.7; *P*=.44). The majority of participants who were lost to follow-up were female participants, single, aged 19 years, and spent an average of 9 hours per day on the web.

**Table 1:** Baseline comparison between intervention and control groups.

Variable			Intervention (n=150)		Control (n=150)		Differences between the conditions				
							Fisher exact test			*P* value	
**Sex, n (%)**								N/A^a,b^			.03^c^
	Male	42 (35)		26 (17.3)							
	Female	108 (65)		124 (82.7)							
**Relationship status, n (%)**								N/A^d^			.86
	Single	131 (87.3)		132 (88)							
	In relationship	19 (12.7)		18 (12)							
Age (years), median (IQR)			19 (0)		19 (0)		–2.2^e^			.03^c^	
Amount of time spent on the web (hours), median (IQR)			8 (6)		8 (7)		–1.1^e^			.28	
Intention to sext score, median (IQR)			16 (6)		16 (7)		–0.3			.73	
Willingness to sext score, median (IQR)			12 (3)		12 (2)		–0.6^e^			.53	
Knowledge of sexting score, median (IQR)			5 (2)		5 (3)		–1.2^e^			.22	
Attitude toward sexting score, median (IQR)			48.5 (27)		50 (24)		–0.9^e^			.37	
Perceived norms toward sexting score, median (IQR)			20 (9)		20 (10)		–0.4^e^			.69	
Prototype perception toward sexters score, median (IQR)			34 (8)		35 (8)		–0.7^e^			.51	

^a^N/A: not applicable.

^b^*χ*^2^_1_=4.9.

^b^Statistically significant at *P*<.05.

^d^*χ*^2^_1_=0.0.

^e^Mann-Whitney test.

### Effectiveness of SIM

Table 2 presents the GLMM results for the effect of group, time, and group and time interaction on the primary outcomes. The findings indicated that after adjusted for age, sex, relationship status, and the amount of time spent on the web, there was a significant difference in the intention to sext (β=–.12; *P*=.002; Cohen *d*=0.23) and willingness to sext (β=–.16; *P*<.001; Cohen *d*=0.40) between the intervention and control groups, which were observed over 3 months. The intervention has resulted in small and moderate effect on intention and willingness to sext, respectively, as shown by Cohen *d.*

Table 3 presents the GLMM results for the effect of group, time, and group and time interaction on the secondary outcomes. The findings showed that after adjusting for age, sex, relationship status, and the amount of time spent on the web, there was a significant difference in the knowledge (β=.12; *P*<.001; Cohen *d*=0.39), attitude (β=–.11; *P*=.001; Cohen *d*=0.31), perceived norms (β=–.07; *P*=.04; Cohen *d*=0.18), and prototype perceptions (β=–.11; *P*<.001; Cohen *d*=0.35) between the intervention and control groups 3 months postintervention. A greater effect of the intervention was observed on the knowledge as compared to other secondary outcomes.

**Table 2:** Effect of the group, time, and group and time interaction on the primary outcomes adjusted for age, sex, relationship status, and the amount of time spent on the web.

Outcomes (measures) and parameters		β	SE	95% CI	*t* test (*df*)	*P* value^a^	Cohen *d*
**Intention to sext**							
	Group	.063	0.083	–0.100 to 0.226	0.764 (857)	.45	0.14
	Time	.084	0.028	0.030 to 0.139	3.028 (857)	.003^b^	0.24
	Group and time	–.120	0.039	–0.196 to –0.043	–3.079 (857)	.002^b^	0.23
**Willingness to sext**							
	Group	.184	0.078	0.030 to 0.337	2.350 (857)	.02^b^	0.46
	Time	.110	0.024	0.063 to 0.157	4.611 (857)	<.001^b^	0.27
	Group and time	–.160	0.033	–0.225 to –0.094	–4.787 (857)	<.001^b^	0.40

^a^Using generalized linear mixed model adjusted for age, sex, relationship status, and the amount of time spent on the web.

^b^Statistically significant at *P*<.05.

**Table 3:** Effect of the group, time, and group and time interaction on the secondary outcomes adjusted for age, sex, relationship status, and the amount of time spent on the web.

Outcomes (measures) and parameters			β		SE		95% CI		*t* test (*df*)		*P* value^a^		Cohen *d*
**Knowledge of sexting**													
	Group	–.141		0.058		–0.254 to –0.027		–2.439 (853)		.02^b^		0.44	
	Time	.071		0.019		0.033 to 0.109		3.689 (853)		<.001^b^		0.22	
	Group and time	.124		0.027		0.071 to 0.177		4.586 (853)		<.001^b^		0.39	
**Attitude toward sexting**													
	Group	.048		0.065		–0.080 to 0.175		0.737 (857)		.46		0.14	
	Time	–.025		0.021		–0.066 to 0.015		–1.222 (857)		.22		0.07	
	Group and time	–.107		–3.721		–0.163 to –0.051		–3.721 (857)		<.001^b^		0.31	
**Perceived norms toward sexting**													
	Group	.005		0.069		–0.131 to 0.140		0.072 (857)		.94		0.01	
	Time	.064		0.021		0.023 to 0.106		0.072 (857)		.003^b^		0.18	
	Group and time	–.062		0.030		–0.120 to –0.004		3.022 (857)		.04^b^		0.18	
**Prototype perception toward sexting**													
	Group	–.025		0.064		–0.151 to 0.102		–0.385 (858)		.70		0.08	
	Time	–.040		–0.018		–0.075 to –0.005		–2.218 (858)		.03^b^		0.13	
	Group and time	–.108		0.025		–0.158 to –0.059		–4.319 (858)		<.001^b^		0.35	

^a^Using generalized linear mixed model adjusted for age, sex, relationship status, and the amount of time spent on the web.

^b^Statistically significant at *P*<.05.

## Discussion

### Principal Findings

To the best of our knowledge, this is the first RCT to evaluate the effectiveness of a theory-based SIM delivered via web-based animated videos on the intention and willingness to sext among diploma students. The secondary outcomes assessed in this study included knowledge, attitude, perceived norms, and prototype perceptions. At the end of 3 months postintervention, the intervention group demonstrated a significant decrease in the intention and willingness to sext as well as a significant improvement in knowledge, attitude, perceived norms, and prototype perceptions among the intervention group compared to the control group.

The changes in the primary and secondary outcomes following the intervention were likely attributed to the PWM constructs and the web-based animated video delivered in this study. Theoretically, sexting is a complex behavior that has been explained by a few theories including PWM. The application of PWM in this intervention allowed a systematic approach to developing the intervention, thus making it easier to develop a storyboard for the animated video. The constructs of PWM, including attitude, perceived norms, and prototype perceptions that have been addressed in the animated videos, led to a significant reduction in the intention and willingness to sext. Furthermore, the use of web-based animated video could have improved the understanding of the module's content since this approach has been recognized as an effective tool for explaining and simplifying complex topics [28]. The combined use of audio and visual learning in the animated video facilitated better retention of information [28] apart from capturing the attention and encouraging the participants to view the entire video. The participants were also given the flexibility and sufficient time to complete viewing all videos. Furthermore, the private YouTube link provided easy accessibility to the participants, thus reducing their hesitancy to view videos that are deemed sensitive topics by many of the local community.

In view of the lack of interventional research on the intention and willingness to sext and sexting-related issues, the findings of this study were compared to other relevant studies that assessed the intention and willingness of risky behaviors. Our findings on the intention to sext were consistent with a study that applied the theory of planned behavior (TPB), the theory of reasoned action (TRA), and PWM in the intervention to measure the intention to commit sexual harassment [37]. Another study that was based on the reasoned action model also reported a significant change in the intention to communicate about sexual topics with a partner after receiving a sexual health intervention [45]. Both studies applied theories with almost similar constructs as PWM that emphasized the role of intention as the determinant of behavior. Furthermore, the findings from this study reinforced the effectiveness of PWM in changing the intention toward specific health behaviors in addition to the TPB and the TRA.

Moreover, there was a significant effect of the intervention on the willingness to sext. This finding was consistent with a study that measured the willingness to assist individuals at risk of sexual assault [46]. Another important finding of this study was that the intervention had a greater effect on the willingness as compared to the intention to sext. This showed that PWM constructs were more effective in influencing the willingness, mainly because they focused more on the role of behavioral willingness or social reactive pathways in deciding on risky behaviors as opposed to the TPB and the TRA, which emphasize the role of behavioral intention [47].

Next, a greater effect of this intervention was observed on the knowledge of sexting. This was consistent with a previous study [34] that applied information, motivation, and behavioral theory to improve HIV-related knowledge with a peer-led education program. Nonetheless, the perceived norms were the least influenced by this intervention, most likely because the participants already regarded sexting as an unacceptable behavior, as most of them were bound by the Islamic religious instructions and traditional Malay culture, which condemn such behavior. Additionally, the technique used in this study, that is, providing the participants with the negative characteristics of sexters, could have contributed to a reduction in the prototype perceptions toward sexters. These results were consistent with other studies that applied PWM [48-51].

### Strengths and Limitations

To date, interventional research on sexting remains scarce. Therefore, this study represents a critical starting point for public health interventions aimed at addressing sexting-related issues in Malaysia. The use of theory-based intervention and web-based animated videos can be seen as a new direction for future public health interventions on SRH topics among young adults, particularly students in HEIs. Moreover, since this study was designed as an RCT, there was a greater internal validity to evaluate the effectiveness of the intervention. In addition, only a few previous RCTs that used a PWM-based intervention reported the effect size of the intervention. We chose to publish the effect magnitude for future comparisons and not depend solely on the *P* value for the significance level. Additionally, the mixed model approach used in this study allowed for the control of the clustering effect and the adjustment of covariates, thereby increasing the internal validity of the study findings.

There are a few limitations to this study. First, the findings were limited to the study population, and they cannot be generalized to the young adult population as a whole. Moreover, the use of the self-reported questionnaire in this study could lead to an information bias. Given the nature of the topic, which involved sexually sensitive questions, the participants might avoid revealing their actual actions or opinions. However, they were reminded that the study aimed to evaluate the effectiveness of the educational material and that no judgment would be made on them. Furthermore, this study did not measure sexting and other multiple-risk behaviors associated with sexting. It would be beneficial to assess the effect of our intervention on the actual sexting behavior, but it would involve a great deal of time and opinions of experts and stakeholders, not to mention the major ethical considerations related to the sensitive nature of the study subject and sexting being considered as punishable under Malaysian law. Finally, it was quite difficult to ensure that all participants in the intervention group adhered to and complied with the intervention, as the animated video delivered via YouTube in this study lacked any monitoring mechanisms.

### Conclusions

The PWM-based intervention module delivered via web-based animated videos in this study was effective in reducing the intention and willingness to sext apart from improving the knowledge of sexting, attitude, perceived norms, and prototype perceptions among diploma students. This intervention can be implemented by relevant agencies involved in the promotion of SRH of young adults in Malaysia. Further research can explore the best ways to increase the effect size of the outcomes, such as providing regular reminders of the contents of the intervention via other web-based approaches.

## Appendix

Multimedia Appendix 1 Summary of the sexting intervention module.

Multimedia Appendix 2 CONSORT (Consolidated Standards of Reporting Trials)-EHEALTH checklist.

## Abbreviations

CONSORT: Consolidated Standards of Reporting Trials
GLMM: generalized linear mixed model
HEI: higher educational institution
PWM: prototype willingness model
RCT: randomized controlled trial
SIM: sexing intervention module
SRH: sexual and reproductive health
TPB: theory of planned behavior
TRA: theory of reasoned action

## Data Availability

The data sets generated during and/or analyzed during this study are available from the corresponding author on reasonable request.
