# Supplementary material for: Effectiveness of a Theory-Based Digital Animated Video Intervention to Reduce Intention and Willingness to Sext Among Diploma Students: Cluster Randomized Controlled Trial
Source: J Med Internet Res. 2023 Oct 20;25:e48968. doi: 10.2196/48968 (PMC10625075; doi:10.2196/48968)
Supplement: Multimedia Appendix 1 [file jmir_v25i1e48968_app1.docx]

**Multimedia Appendix 1**

**Summary of the Sexting Intervention Module (SIM)**

| **Section** | **PWM Constructs** | **Aim** | **Contents** | **Duration** |
| --- | --- | --- | --- | --- |
| Section 1  Sexting and Z generation | - | To increase knowledge of sexting among participants. | i. Provide information about the sexting trend among the Z generation and the reasons why it is common among them. | 2.4 minutes. |
| Section 2  What are the implications of sexting? | Attitude | To improve attitude toward sexting.  To reduce intention and willingness to sext among participants. | i. Provide information on the negative consequences of sexting from four aspects which are sexual and reproductive health, cyberbullying, mental health, and legality. | 9.3 minutes. |
|  |  |  | ii. Provide real world negative consequences of sexting to the individuals based on the reports by local media. |  |
| Section 3  Who are behind sexting? | Prototype perception | To improve prototype perception.  To reduce intention and willingness to sext among participants. | i. Provide information about the characteristics or personality of individuals who are associated with sexting behavior. | 3 minutes. |
| Section 4  What others’ opinion? | Perceived norm | To reduce perceived norm, intention, and willingness to sext among participants. | i. Provide opinions of parents and other young adults on individuals who are involved in sexting. | 3.2 minutes. |
| Section 5:  What you can do. | Perceived norm  Prototype perception | To reduce perceived norm, prototype perception, intention, and willingness to sext among participants | i. Provide information on potential scenarios that might lead to sexting.  ii. Provide information on potential barriers of resisting sexting.  iii. Provide skills to resist sext requests by others and information on where to get help. | 5.4 minutes. |
